# 5-methylcytosine RNA methyltransferases and their potential roles in cancer

**DOI:** 10.1186/s12967-022-03427-2

**Published:** 2022-05-13

**Authors:** Mingyang Li, Zijia Tao, Yiqiao Zhao, Lei Li, Jianyi Zheng, Zeyu Li, Xiaonan Chen

**Affiliations:** Department of Urology, Shengjing Hospital of China Medical University, No. 36 Sanhao Street, Heping District, Shenyang, 110004 Liaoning People’s Republic of China

**Keywords:** 5-methylcytosine, Cancer, RNA methylation, RNA methyltransferases, Molecular mechanisms, Prognosis

## Abstract

In recent years, 5-methylcytosine (m^5^C) RNA modification has emerged as a key player in regulating RNA metabolism and function through coding as well as non-coding RNAs. Accumulating evidence has shown that m^5^C modulates the stability, translation, transcription, nuclear export, and cleavage of RNAs to mediate cell proliferation, differentiation, apoptosis, stress responses, and other biological functions. In humans, m^5^C RNA modification is catalyzed by the NOL1/NOP2/sun (NSUN) family and DNA methyltransferase 2 (DNMT2). These RNA modifiers regulate the expression of multiple oncogenes such as fizzy-related-1, forkhead box protein C2, Grb associated-binding protein 2, and TEA domain transcription factor 1, facilitating the pathogenesis and progression of cancers. Furthermore, the aberrant expression of methyltransferases have been identified in various cancers and used to predict the prognosis of patients. In this review, we present a comprehensive overview of m^5^C RNA methyltransferases. We specifically highlight the potential mechanism of action of m^5^C in cancer. Finally, we discuss the prospect of m^5^C-relative studies.

## Background

Post-transcriptional modifications have become an important field of research with more than 170 RNA modifications being identified [1]. These modifications can significantly affect the biogenesis and function of coding and non-coding RNAs to mediate metabolism and play a regulatory role in the occurrence and progression of diseases. 5-methylcytosine is observed in a wide range of RNAs; it is the most abundant in tRNA and rRNA but has also been identified in mRNA and other noncoding RNAs [2]. According to liquid chromatography-tandem mass spectrometry analysis, the methylation level of m5C is estimated to be 0.02–0.09% [3]. Currently, m5C detection methods are divided into three groups based on their principles: (1) immunoprecipitation-based sequencing, (2) chemical-dependent sequencing, and (3) third-generation sequencing based on electronic current signals (extensively reviewed in [4]). Although numerous studies are being conducted on m5C modification, its molecular mechanism and role in the pathophysiology of an organism is largely unknown.

Similar to m6A methylation, the enzymes regulating m^5^C levels of RNAs can be functionally categorized as “writers,” “erasers,” and “readers”. Methyltransferases, or writers, can install m^5^C on RNA. NSUN1-7 and DNMT2 have been well documented as m^5^C writers. Erasers or m^5^C demethylases, such as alpha-ketoglutarate-dependent dioxygenase ABH1 (ALKBH 1) and ten-eleven translation family proteins (TET), are known to remove m^5^C modifications from RNAs. The former can oxidize m^5^C of tRNA into 5-formylcytosince (f^5^C) in the mitochondria [5, 6], while the latter can oxidize m^5^C of mRNA into 5-hydroxymethylcytosine (hm^5^C) [7, 8]. Binding proteins that recognize m^5^C in RNAs are called readers. Known readers include RNA and export factor-binding protein 2 (ALYREF) [9] and Y-box-binding protein 1 (YBX1) [10], where the protein Lin-28 homologous B (LIN28B) is also reported to possess the characteristics of a reader [11].

At present, writers of m^5^C have been studied exhaustively thereby giving a better functional clarity in different processes. This review focuses on the effects of m^5^C methyltransferases in molecular and cellular functions and their potential roles in cancer.

## Main body

### RNA Methyltransferases mediating m^5^C

**Fig. 1:**
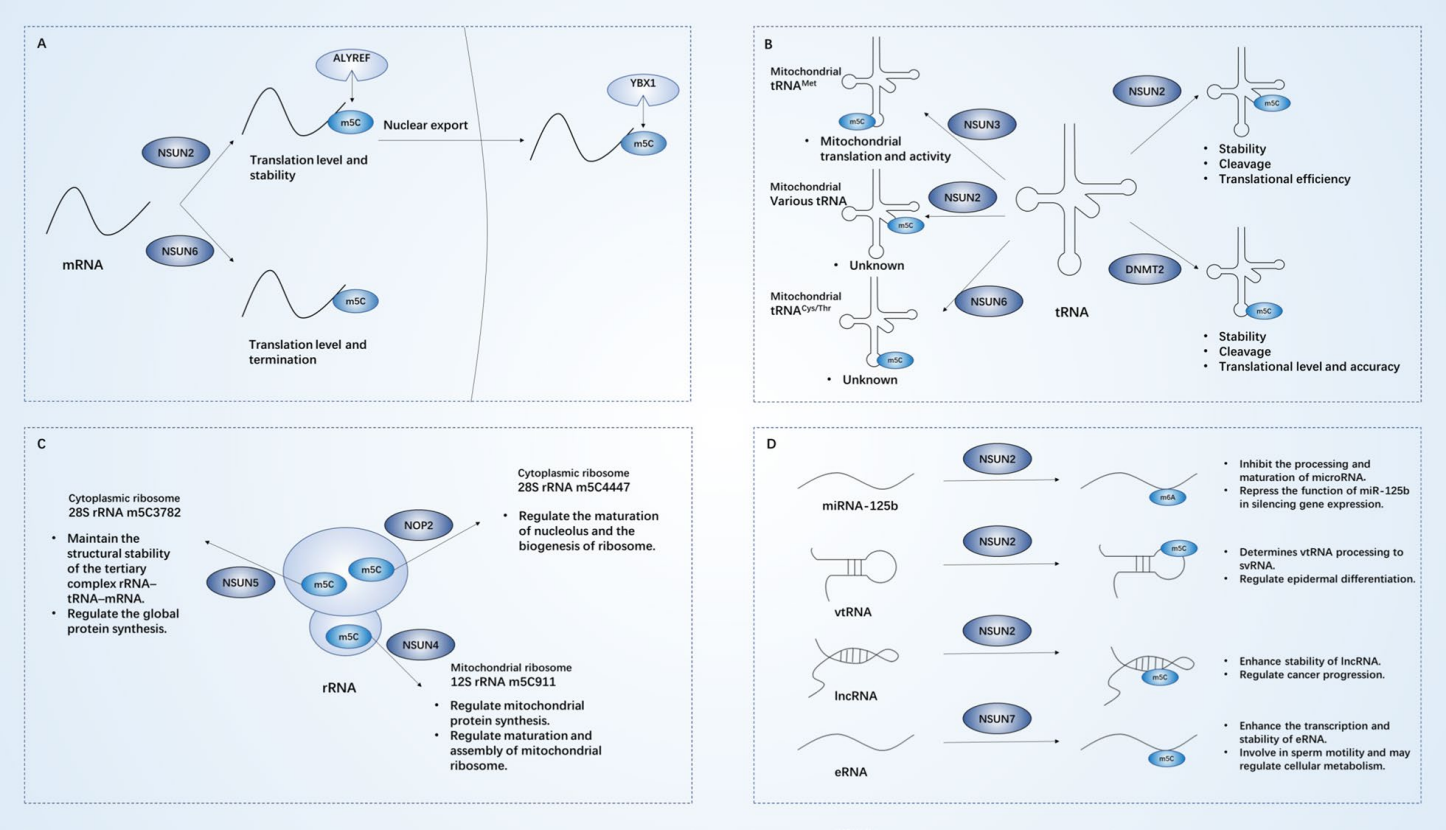
Molecular mechanism and functions of m^5^C methyltransferases. m^5^C modification of **A** mRNA, **B** tRNA, **C** rRNA, and **D** non-coding RNA such as lncRNA, microRNA, vtRNA, and eRNA. m^5^C modification of RNA can modulate the molecular functions of RNAs and mediate the regulation of cellular metabolism

m^5^C methylation of human RNA is mainly catalyzed by the NOL1/NOP2/sun family and DNMT2, important for RNA stability and functionality. Methyltransferases transfer the methyl groups to cytosine through S-adenosylmethionine as a methyl donor to form m^5^C. Different cellular compartments possess the resident enzymes that bring about the modification. In the nucleus, m^5^C of mRNA, tRNA, 28S rRNA, and non-coding RNAs is mainly methylated by NSUN2, NSUN5, NSUN6, NSUN7, and NOP2. In the mitochondria, NSUN2 and NSUN3 methylate tRNA, and NSUN4 methylates 12S rRNA that promotes mitochondrial ribosome assembly (Table 1 and Fig. 1). The molecular mechanisms of m^5^C RNA methyltransferases and their biological functions are detailed below.

**Table 1:** Molecular mechanisms and cellular function of m5C enzymes

Regulator	Target RNA(s)	Modification installed	Biological functions	Mechanisms	Refs.
NSUN1/NOP2	28S rRNA	m^5^C4447	Unknown	Unknown	[12]
	telomerase RNA component	Unknown	Maintain the proliferation of human tumor cells	Activates and regulates cyclin D1 gene transcription	[16]
	TAR RNA in HIV-1 virus	Unknown	Inhibit viral transcription and increase its latency	Competes with Tat protein binding to TAR	[17]
NSUN2	mRNA	various	Enhance cellular migration	Promotes the translation and export of ATX mRNA	[9] [72]
			Regulate cellular senescence and proliferation	Regulates the translation of SHC, CDK1, p21, and p27 mRNAs	[33–36]
			Mediate vascular inflammation and allograft arteriosclerosis	Upregulates the expression of ICAM-1 mRNA	[32]
	nuclear or cytoplasmic tRNA	m^5^C48/49/50	Regulate cellular response to external stress stimuli	Regulates the stability and cleavage of cytoplasmic tRNAs	[40, 41]
			Regulate protein synthesis of cells	Promotes stability and translational efficiency of tRNA	[42, 43]
	mitochondrial tRNA	m^5^C48/49/50	Unknown	Unknown	[44]
	vtRNA	various	Regulate epidermal differentiation	Determines vtRNA processing to svRNA	[47, 48]
	microRNA-125b	m^6^A9,15	Repress the function of silencing gene expression	Inhibits processing and maturation of microRNA	[45, 46]
	lncRNA	various	Regulate the progression of cancer	Enhances lncRNA stability	[49, 50]
NSUN3	mt-tRNA^Met^	m^5^C34	Promote mitochondrial translation and regulate mitochondrial activity	Enables mt-tRNA^Met^ to recognize AUA and AUG codons	[6, 75, 76]
NSUN4	mt-12S rRNA	m^5^C911	Regulate mitochondrial protein synthesis	Regulates maturation and assembly of mt-ribosome	[82–91]
NSUN5	28S rRNA	m^5^C3782	Regulate global protein synthesis and proliferation of cells	Maintains the structural stability of the tertiary complex rRNA–tRNA–mRNA	[94, 95]
	Tpm1 mRNA?	Unknown	Maintain the normal morphogenesis of the cardiac outflow tract	Regulates the translation of Tpm1 mRNA	[99]
NSUN6	tRNA^Cys/Thr^	m^5^C72	Unknown	Unknown	[104]
	mRNA	various	Suppress the development of pancreatic cancer	May enhance expression of CDK10 mRNA	[154]
			Regulate bone metastasis of breast cancer	Affects the kinase activity of MST1 and activate YAP	[105]
NSUN7	eRNA	Unknown	May regulate cellular metabolism	Enhances the transcription and stability of eRNA	[106]
DNMT2	tRNA^Asp^	m^5^C38	Regulate cellular response to external stress stimuli	Regulates tRNA stability and mediates its cleavage	[43, 116–118]
			Regulate cellular differentiation and protein synthesis accuracy	Discriminates near-cognate codons	[119]
	mRNA?	Unknown	Affect the migration and invasion of HEK293 cells	Unknown	[120]

### NOP2

NOP2 (Nucleolar protein 2, also termed NSUN1) methylates human 28S rRNA cytosine at position 4447 (C4447) [12]. It is necessary for the development of mammalian embryos by regulating nucleolar maturation at the preimplantation stage leading to blastocyst formation, and in ribosome biogenesis. Notably, rRNA processing requires the presence rather than the m^5^C modification activity of NOP2 [13, 14]. In addition, NOP2 promotes cell proliferation during nerve tissue regeneration [15]. In human tumor cells, NOP2 is shown to combine with the telomerase RNA component (TERC) via its rRNA methyltransferase domain, thereby activating and regulating cyclin D1 gene transcription, which maintains cell proliferation [16]. In HIV-1 virus, NOP2 binding to TAR RNA at the 5'-long terminal repeat (LTR) leads to addition of m^5^C, thereby inhibiting viral transcription and promoting its latency by competing with the TAT protein [17].

NOP2 is upregulated by microRNA PVT1 to promote hepatocellular carcinoma (HCC) proliferation and prostate cancer metastasis [18, 19]. It also presents aberrant expression in several cancers, such as renal clear cell carcinoma, lung adenocarcinoma, colorectal cancer, and low-grade glioma, providing risk signatures associated with m5C methylation that can aid in the determination of patient prognosis [20–26].

### NSUN2

NSUN2, predominantly located in the nucleus, is a direct target of c-MYC, which recruits nucleolar and spindle-associated protein (NuSAP) to stabilize the mitotic spindle in fast-dividing cells and adds m^5^C to mRNA and several noncoding RNAs [27, 28].

In mRNAs, m^5^C sites are distributed throughout the genome and are most frequently located in C-G rich regions. These sites are enriched in untranslated regions (UTRs) of mRNA, especially in the vicinity of the binding region of the Argonaute protein within the 3' UTRs [9, 29, 30]. The distribution of m^5^C sites in translation sequence (CDS) has not yet been determined. According to Tao Huang et al. [29], m^5^C sites had the lowest density in CDS; this view was not supported by Xin Yang et al. [9], who indicated that m^5^C sites were also abundant in regions immediately downstream of translation initiation sites. NSUN2-dependent m5C sites tend to be located at the 5' end of a stem-loop structure with a 3' G-rich triplet (3' CNGGG) motif as the specific structure and a sequence preference for NSUN2. This specific motif has been observed in multiple human and mouse tissues, demonstrating that NSUN2 is a major mRNA methyltransferase. Notably, another specific motif 3' CTCCA, which has also been detected in multiple tissues, has been identified as a specific sequence of NSUN6, another m^5^C methyltransferase of mRNA [29]. m^5^C modulates mRNA export through specific recognition of the mRNA export adaptor ALYREF [9] and regulates mRNA stability and translation. NSUN2 methylates interleukin-17A (IL-17A) mRNA to mediate the hyperhomocysteinemia (HHcy)-induced upregulation of IL-17A expression and promotes its translation in T lymphocytes. [31] NSUN2 upregulates the expression of intercellular adhesion molecule-1 (ICAM-1) by adding m^5^C to ICAM-1 mRNA, which affects vascular inflammation and allograft arteriosclerosis [32]. Moreover, NSUN2-mediated mRNA modification regulates the translation of various mRNAs such as SHC, cyclin-dependent kinase 1(CDK1), p21, and p27, to promote or delay cellular senescence [33–36]. Interestingly, m^5^C and m6A modifications of p21 mRNA facilitate each other and together they affect protein expression [35]. It has also been reported that NSUN2 introduces m6A in the 3' UTR of p16 mRNA to stabilize its structure and promote its expression under oxidative stress [37]. These findings indicate a novel methylation modification pattern via interaction with various RNA methyltransferases. Furthermore, NSUN2 appears to act as a double-edged sword in the regulation of mRNA stabilization. In bladder cancer, the cytoplasmic protein YBX1 recognizes the NSUN2-dependent m5C site located on the 3'UTR of heparin-binding growth factor (HDGF) mRNA and recruits ELAV-like RNA-binding protein 1 (ELAV1) to improve its stability. This specific recognition is attributed to the cold shock domain (CSD) of YBX1 [10]. LIN28B also has a similar structure [38] and stabilizes growth factor receptor-bound protein 2 (GRB2) mRNA in an NSUN2-dependent manner in esophageal squamous cell carcinoma (ESCC), thus indicating that it is a potential m5C reader [11]. Additionally, in gastric cancer (GC), NSUN2 modifies the 3'UTR of cyclin-dependent kinase inhibitor 1C (CDKN1C, p57^Kip2^) mRNA to repress its stability, decreasing the half-life of p57^Kip2^ mRNA [39].

NSUN2 also modifies multiple cytoplasmic tRNAs with m^5^C, mediating cleavage and modulating stability to participate in the cell stress response. Exposure to oxidative stress effectively inhibits NSUN2, causing a decline in methylation at specific tRNA sites thereby resulting in increased angiogenin-mediated endonucleolytic cleavage of tRNA and accumulation of 5' tRNA-derived small RNA fragments (5' tRFs). The accumulation of 5' tRFs reduces the rate of protein translation and activates the stress pathway, leading to a decrease in cell size and increased apoptosis in the cortex, hippocampus, and striatal neurons in response to external stress stimuli [40, 41]. Modification of tRNA by NSUN2 also affects its translation efficiency. Knockout of NSUN2 in mouse neurons results in glycine-specific translation deficiency [42, 43]. In addition, NSUN2 methylates mitochondrial tRNA, however, inactivation of NSUN2 had no profound effect on the stability of mitochondrial tRNA and oxidative phosphorylation in differentiated cells [44].

It also methylates various ncRNAs to regulate their function. NSUN2 mediated methylation of miRNA-125b inhibits its processing and function in gene silencing [45, 46]. Notably, NSUN2 methylates miRNA-125b in an m6A manner rather than m^5^C. Vault RNA m^5^C modification by NSUN2 determines its processing to svRNA, which participates in the regulation of epidermal differentiation [47, 48], while its processing to lncRNA promotes tumorigenesis and aggression in several cancers [49, 50].

Owing to the extensive list of targets, NSUN2 plays a significant role in several processes including modulating cell functions in proliferation [34], stress response and metabolism [40, 41], migration and differentiation [51], and senescence processes [33–36]. It is associated with many diseases such as autism spectrum disorder [52], depression [42], Dubowitz syndrome [53, 54], intellectual disability [55–57], and is differentially expressed in a variety of cancers [20, 22, 58–68]. In recent years, several studies have explored its molecular mechanisms, constructed prognostic models, and attempted to find new targets for cancer treatment [11, 39, 46, 49, 50, 69–74]. Currently, studies regarding the regulation of NSUN2 in terms of biological function and cancer mechanism focus on its modification of mRNA. However, the pathway underlying the modifications of ncRNA induced by NSUN2 to interact with mRNA and proteins needs to be further investigated and explored. Moreover, although not yet discussed, the mechanism by which tRNA cleavage affects cellular stress responses may have significant potential for furthering the understanding of cancer.

### NSUN3

In the mitochondria, NSUN3 mediates mt-tRNA^Met^ methylation of cytosine at position 34 (C34) into m^5^C34 which is further oxidized by ALKBH1/ABH1 into f5C34 [75, 76]. f^5^C34 enables mt-tRNA^Met^ to recognize AUA and AUG codons encoding methionine [6]. NSUN3 knockout and mutant cells show decreased mitochondrial protein synthesis and reduced oxygen consumption, resulting in mitochondrial dysfunction [6]. A biallelic missense mutation in NSUN3 led to early onset mitochondrial encephalomyopathy and seizures [77]. Mutations in the NSUN3 gene may cause damage to the nervous system. Trixl et al. demonstrated the effect of inactivation of NSUN3 on the self-renewal and differentiation potential of mouse embryonic stem cells [78].

NSUN3 has been reported to be upregulated in several cancers, [20, 24, 79] and is associated with immune cell infiltration [79]. Its overexpression may play a regulatory role in sensitizing the cells against the chemotherapy drugs, thereby affecting patient prognosis [80, 81].

### NSUN4

NSUN4 is a bifunctional protein playing a role in methylation of 12S rRNA at cytosine 911 (m^5^C911) [82–85], and interacting with MTERF4 to promote monomer assembly [82–91]. Though the mechanism is still unclear, m^5^C911 may cooperate with nearby m^4^C909 and other rRNA modifications to stabilize 12S rRNA folding, thereby facilitating mt-ribosome assembly [85].

NSUN4 expression affects embryonic development and mitochondrial protein synthesis. Germline knockout of the NSUN4 gene in mouse is embryonically lethal, and the conditional knockout in the heart is shown to interrupt mitochondrial protein translation, leading to impaired respiratory complex formation [92].

NSUN4 is aberrantly expressed in lung adenocarcinoma, hepatocellular carcinoma, and clear cell renal cell carcinoma and may be utilized to predict prognosis [20, 23, 79, 93].

### NSUN5

NSUN5 introduces m^5^C at C3782 in the human 28S ribosomal RNA. Mammalian NSUN5 deficiency alters the ribosome affecting total protein synthesis impinging on cell size and proliferation [94]. This can be attributed to the maintenance of the tertiary rRNA-tRNA-mRNA complex due to m^5^C3782 [95].

NSUN5 also affects the development and function of the nervous system. Its deletion is associated with Williams–Beuren syndrome (WBS) [96–98]. The expression of NSUN5 is critical for cerebral cortex development. It controls the migration of neocortical neurons by regulating the radial glial scaffold of retinal ganglion cells [98]. NSUN5 deficiency disturbs the laminar organization of neocortical neurons and the development of pyramidal cells. This causes reduced proliferation of oligodendrocyte precursor cells and hypomyelination leading to agenesis of the corpus callosum (CC) and dysfunction of the NMDA receptor (NMDAr) in hippocampal pyramidal cells [96, 97]. Moreover, in the cardiovascular system, NSUN5-mediated m^5^C modification is essential for maintaining the expression of Tpm1, which is an essential gene for normal cardiac outflow tract (OFT) morphogenesis, suggesting the involvement of NSUN5 in the tetralogy of Fallot (TOF) [99].

NSUN5 is significantly upregulated in head and neck squamous cell carcinoma (HNSCC) [100] and acts as a promoter of colorectal cancer (CRC) by triggering cell cycle arrest.[101]. Its epigenetic inactivation is observed in gliomas and exhibits tumor-suppressive characteristics [95].

### NSUN6

NSUN6 has a strong substrate specificity for mRNA, mainly targeting the 3' UTR at the consensus sequence motif CTCCA located in the loops of hairpin structures to install m^5^C modifications, rather than the 3' CNGGG motif targeted by NSUN2 [29, 102]. The NSUN6-targeted CTCCA motif marks the translational termination. The methylated hairpin structure at 3'UTR is likely responsible for translational termination, but there is no evidence to confirm this view [103]. In human HEK and H9 cell lines, NSUN6 primarily targets mRNAs encoding RNA- and protein-binding proteins. NSUN6-mediated m^5^C modification enhances mRNA abundance and translation efficiency. [102]. It also methylates cytosine 72 (C72) at the 3′-end receptor stems of tRNA^Cys^ and tRNA^Thr^. Target recognition depends on the presence of a 3′-CCA tail [104].

In tumors derived from tissues with high NSUN6 expression, NSUN6 mRNA levels are downregulated. In contrast, when tumors were derived from NSUN6 low-expressing tissues, there was no difference in RNA levels [102]. NSUN6 has also been shown to inactivate macrophage stimulating 1 (MST1) and activate yes-associated protein (YAP) target genes in breast cancer through m^5^C modification, thereby triggering osteoclast differentiation and bone metastasis [105]. As these are m5C methyltransferases of mRNA, correlations between NSUN2 and NSUN6 have been analyzed using bioinformatics, which have shown them to be positively correlated, uncorrelated and negatively correlated in renal cancer [23], triple-negative breast cancer [59], and cutaneous melanoma [24], respectively. However, all studies conducted to date have failed to provide direct evidence to support the correlation between the two enzymes. Furthermore, no reader has been detected to recognize NSUN6-dependent m5C sites on mRNA, which hinders further understanding of the regulatory role of NSUN6 in cell metabolism and cancer progression.

### NSUN7

The interaction between NSUN7 and peroxisome proliferator-activated receptor-gamma coactivator 1 alpha (PGC-1α) promotes transcription of fasting related genes. Meanwhile, NSUN7 enhances the stability of eRNAs through m^5^C modification and may be involved in the regulation of cell metabolism [106].

Moreover, NSUN7 mutation can lead to impaired sperm quality and infertility [107, 108]. This may be caused by the transversion mutation of exon7, thereby affecting protein structure and ligand-binding site [109]. However, this mutation is not associated with asthenospermia in Han Chinese men [110]. In addition, NSUN7 is also correlated with mental disorders [111] and is used in the prognosis of patients with Ewing sarcoma, low-grade glioma, and prostate cancer [112–114].

### DNMT2

Compared with other DNA methyltransferases, such as DNMT1, DNMT3a, and DNMT3b, DNMT2 exclusively consists of the C-terminal catalytic domain but lacks the N-terminal regulatory domain. [115] DNMT2 (also termed TRDMT1) does not possess DNA catalytic activity but introduces m^5^C38 into tRNA^Asp^ (GUC) [116].

The m^5^C modification mediated by DNMT2 improves tRNA stability, where tRNA^Asp^ is protected from ribonuclease cleavage during the heat shock response in Drosophila and is protected from fragmentation in mice [43, 117]. Moreover, DNMT2 influences the expression and precision of protein synthesis via m^5^C. DNMT2-mediated tRNA^Asp^ m^5^C38 regulates the translation of proteins containing poly-Asp sequences. Mouse aspartyl-tRNA synthetase shows a four-to-five-fold preference for C38 methylated tRNA^Asp^ [118]. DNMT2 also ensures precise peptide synthesis through the discrimination of near-cognate codons and is necessary for cell differentiation and protein synthesis [119]. It also participates in the regulation of mRNA methylation and affects the migration and invasion of HEK293 cells [120].

DNMT2 plays a regulatory role in the cellular stress response. Under stress conditions, DNMT2 localizes to cytoplasmic stress granules and RNA-processing bodies [121, 122]. DNMT2 silencing results in enhanced oxidative stress, genomic instability, permanent inhibition of cell proliferation, diminished telomere length and telomerase activity, global RNA hypermethylation, and upregulation of multiple miRNAs related to proliferation and tumor suppression [123, 124].

### Potential roles of m^5^C RNA methyltransferases in cancer

**Fig. 2:**
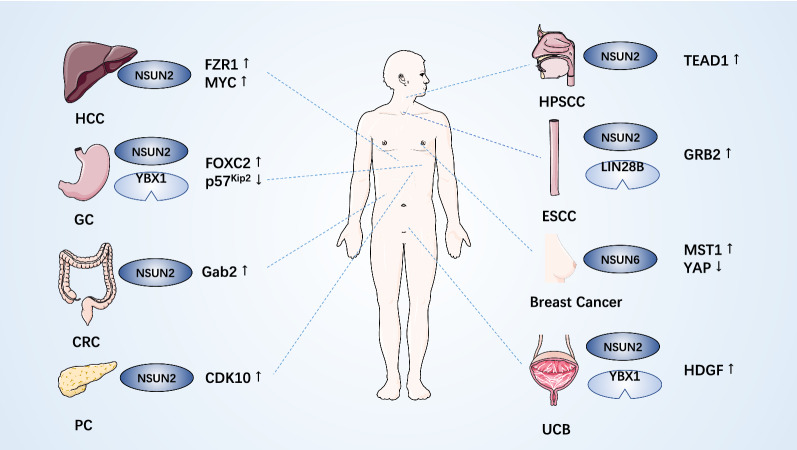
Potential roles of m^5^C methyltransferases in human cancer. The potential roles of m^5^C methyltransferases in cancer are reflected via the regulation of tumor-related gene expression

m^5^C methyltransferases, especially NSUN2, regulates substrate levels by catalyzing m^5^C modification of target RNA to mediate the crosslinking of a series of oncogenic or antitumor factors, thus affecting tumorigenesis and cancer progression. Here, we elaborate on the aberrant expression and corresponding mechanism of m^5^C methyltransferase in cancer (Table 2 and Fig. 2).

**Table 2:** Roles of m5C enzymes in cancer

Cancer type	Enzyme and relative RNA	Aberrant expression	Target	Effect of targets	Roles in cancer	Refs.
HCC	NSUN2	Upregulation	FZR1 mRNA	Enhances stability	Enhances the growth of HCC cells and tumors	[58]
			H19 lncRNA	Enhances stability	Promotes proliferation, migration, invasion, and angiogenesis and inhibits apoptosis	[49]
	NOP2	Upregulation	Unknown	–	Promotes carcinogenesis, cell proliferation, and stem cell-like properties	[125]
	NSUN4	Upregulation	Unknown	–	Unknown	[93]
Gastric Cancer	NSUN2	Upregulation	FOXC2 mRNA	Enhances stability	Facilitates proliferation, migration, and invasion	[69]
			p57kip2 mRNA	Represses expression	Promotes the proliferation of GC cells	[39]
GIST	DNMT2	Upregulation	Unknown	–	Unknown	[142]
CRC	circNSUN2	Upregulation	HMGA2 mRNA	Enhances stability	Promotes liver metastasis	[73]
			miR-181a-5p	Repress expression	Enhances ROCK2 expression to promote proliferation and migration and inhibits apoptosis	[46, 70]
	NSUN2	Upregulation	miR-125b	Inhibits processing	Enhances Gab2 expression to promote cell migration	[46]
	NSUN5	Upregulation	Unknown	–	Promotes proliferation and maintains cell cycle	[101]
Glioma	NSUN5	Downregulation	28S rRNA	Deletes m^5^C3782	Changes ribosome structure, repressing global protein synthesis	[95]
					Activates stress adaptive translational programs	[95]
	NSUN3, DNMT2 and NOP2	Upregulation	Unknown	–	Unknown	[22]
Breast Cancer	NSUN2, NOP2	Upregulation	Unknown	–	Promotes proliferation, migration, invasion, and tumorigenicity of cancer cells	[67, 74]
	NSUN6	Downregulation	MST1 Protein	Inactivation	Activates YAP to promote tumor cell proliferation and bone metastasis	[105]
UCB	NSUN2	Upregulation	HDGF mRNA	Enhances stability	Promotes invasion and metastasis	[10]
Prostate Cancer	DNMT2	Upregulation	Unknown	–	Unknown	[148]
	NOP2	Upregulation	Unknown	–	Promotes metastasis and invasion through the EMT pathway	[18, 19]
ccRCC	NSUN6, NSUN5, NSUN2, NOP2 and DNMT2	Upregulation	Unknown	–	Unknown	[20, 21, 23]
	NSUN3, NSUN4 and NSUN7	Downregulation	Unknown	–	Unknown	[20, 23]
Leukemia	NSUN3, DNMT2	Undetermined	hnRNPK	Enhances integrity	Involved in forming 5-AZA-sensitive active chromatin structure	[81]
	NOP2	Undetermined	RNA-pol-II	Unknown	Involved in forming 5-AZA-resistant active chromatin structure	[81]
Gallbladder Carcinoma	NSUN2	Upregulation	Unknown	–	Promotes growth and tumorigenesis	[149]
LUSC	NSUN3, NSUN4	Upregulation	Unknown	–	Unknown	[79]
Lung adenocarcinoma	NOP2	Upregulation	Unknown	–	Involved in poor differentiation	[26]
CM	NOP2, NSUN5	Upregulation	Unknown	–	Unknown	[24]
	NSUN6, NSUN7	Downregulation	Unknown	–	Unknown	[24]
ESCC	NSUN2	Upregulation	GRB2 mRNA	Enhances stability	Activates PI3K/Akt and ERK/MAPK signaling	[11]
			NMR lncRNA	Unknown	Promotes metastasis and invasion and enhances cisplatin resistance	[50]
HNSCC	NSUN2	Upregulation	Unknown	–	Unknown	[63, 66]
HPSCC	NSUN3	Upregulation	TEAD1 mRNA	Enhances expression	Enhances proliferation and invasion	[71]
PC	NSUN6	Downregulation	CDK10	Enhances expression	Inhibits proliferation	[154]

### Hepatocellular carcinoma

In hepatocellular carcinoma (HCC), the mutation frequency of m^5^C regulatory genes is high, and the dysregulation of m^5^C related genes is associated with higher stages of HCC [93]. In HCC cells, lncRNA-PVT1 combines with NOP2 to upregulate its expression via stability enhancement. The hPVT1/NOP2/cell cycle pathway promotes carcinogenesis, cell proliferation, and stem cell-like properties. Targeting this pathway may have therapeutic potential in HCC [125].

The transcript level of NSUN2 is upregulated in HCC cells, which promotes proliferation, migration, invasion and angiogenesis, and inhibits apoptosis of HCC cells [49, 58]. NSUN2 increases the stability of fizzy-related-1 (FZR1) mRNA thereby modulating FZR1 expression, leading to enhanced growth of HCC cells and tumors [58]. FZR1 is a coactivator of the anaphase-promoting complex or cyclosome [126]. As an E3 ubiquitin ligase, FZR1 regulates mitosis and the G1 phase of the cell cycle [127]. Recently, FZR1 has been found to play a regulatory role in colorectal cancer [126], breast cancer [128], B-cell acute lymphoblastic leukemia [129], and multiple myeloma [130]. NSUN2 silencing inhibits FZR1, inducing cell cycle arrest and increased apoptosis in HCC cells. Notably, NSUN2-KO cells inhibit the expression of FZR1 in gastric cancer cells, which is consistent with HCC [39]. However, the role of NSUN2-FZR1 in migration and invasion in HCC is not clear [58]. Moreover, NSUN2 introduced m^5^C986 at the H19 lncRNA to enhance its stability. NSUN2 deficiency significantly reduces the half-life of H19 RNA [49]. m^5^C modification of H19 RNA enhances its specific binding to the tumor protein G3BP1, which binds to MYC mRNA and promotes its decay [131]. In contrast, m^5^C-modified H19 RNA may compete with MYC mRNA to bind to G3BP1, leading to MYC accumulation and promoting the development of HCC cells. High levels of H19 expression and m^5^C-modification are related to poor differentiation in HCC [49].

In addition, NSUN4 and m^5^C reader ALYREF are upregulated in HCC and are associated with poor prognosis [93].

### Gastrointestinal cancer

Bioinformatics analysis showed that the expression of all regulators of m^5^C, except NSUN6, was significantly upregulated from pathological stages I to IV in gastrointestinal (GI) cancer and, except NSUN7, was associated with shorter overall survival (OS). m^5^C regulators have the greatest impact on ErbB and PI3K-Akt signaling pathways, and BSK3B is an important potential target of the m^5^C regulators [61].

Among GI tumors, NSUN2 has the highest mutation rate [61]. In gastric cancer (GC) cells, a small ubiquitin-like modifier (SUMO)-2/3 interacts with the NSUN2 protein to promote its stability and mediate its import into the nucleus. NSUN2 promotes tumor progression through both m^5^C-dependent and -independent pathways [60]. NSUN2 is recruited by the lncRNA forkhead box protein C2 (FOXC2)-AS1 to modify FOXC2 mRNA in an m^5^C-dependent manner. The m^5^C reader YBX1 combines with methylated FOXC2 mRNA to enhance its stability, thereby facilitating the proliferation, migration, and invasion of GC cells [60]. FOXC2 is an oncogene, overexpressed in multiple cancers promoting cell proliferation and inducing epithelial-mesenchymal transition (EMT) [132–136]. Moreover, NSUN2 destabilizes the p57^Kip2^ transcript by introducing m^5^C modifications in the 3-UTR of p57^Kip2^ mRNA, thereby repressing its expression and promoting the proliferation of GC cells [39]. p57^kip2^ is a CDK inhibitor of the CIP/Kip family that participates in several biological processes [137, 138]. It functions as an antitumor factor in gastric cancer and is down-regulated in multiple cancers [139–141]. In addition, in NSUN2-KO GC cells, PIK3R1 and PCYT1A mRNAs were downregulated, with diminished m^5^C peaks. Bioinformatics analysis of the TCGA data set showed that high expression of phosphoinositide-3-kinase regulatory subunit 1 (PIK3R1) and phosphate cytidylyltransferase 1A (PCYT1A) was associated with a poor prognosis of GC [60].

In addition, DNMT2 is significantly overexpressed in adult gastrointestinal stromal tumors (GISTs) compared to adjacent non-tumor tissues [142].

### Colorectal cancer

Circular RNAs (circRNAs) are a class of non-coding RNAs produced by back-splicing [143]. Circ NSUN2, NSUN2, and NSUN5 are upregulated in CRC and promote its progression. Overexpression of circNSUN2 promotes the metastasis, migration, and proliferation of CRC cells and inhibits tumor cell apoptosis. Mediated by YTH domain-containing 1 (YTHDC1) in an m6A-dependent manner, circNSUN2 is exported from the nucleus to the cytoplasm, where high levels of circNSUN2 enhance the stability of high-mobility group AT-hook 2 (HMGA2) mRNA by forming a circNSUN2/ insulin like growth factor 2 mRNA binding protein 2 (IGF2BP2)/HMGA2 RNA–protein ternary complex, resulting in liver metastasis (LM) of CRC [73]. Moreover, as a miRNA sponge, circNSUN2 targets miR‑181a‑5p and downregulates its expression. The oncogene Rho-associated coiled-coil containing protein kinase 2 (ROCK2) is downregulated by miR‑181a‑5p. The repression of the negative regulation of miR‑181a‑5p on ROCK2 mediated by circNSUN2 promotes the proliferation and migration of CRC cells and inhibits their apoptosis [70]. In addition, circNSUN2 targets miR-296-5p and is downregulated by alopperine (ALO), which upregulates the abnormally low expression of miR-296-5p in CRC. miR-296-5p binds to STAT3 and inhibits its expression, thus inhibiting the proliferation and promoting apoptosis of CRC cells. CircNSUN2 silencing inhibits CRC cell proliferation, which can be neutralized by a miR296-5p inhibitor. ALO regulates the circNSUN2/miR-296-5p/STAT3 pathway to prevent colorectal cancer [144].

In colorectal cancer specimens, NSUN2 is activated by protein activated receptor 2 (PAR2) and methylated pre-mir-125b in an m6A-dependent manner to interfere with its processing, thereby reducing the level of miR-125b. Grb associated-binding protein 2 (Gab2) mediates cell migration, which is repressed by miR-125b. The suppression of miR-125b enhances Gab2 expression, thereby promoting cell migration [46].

NSUN5 is upregulated in CRC tissues and cells. NSUN5-KO mice showed a significant reduction in cell proliferation and induced cell cycle arrest. GSEA suggested that NSUN5 may promote the proliferation of colorectal cancer cells through the Rb-CDK signal transduction pathway [101].

### Glioma

In low-grade gliomas, several m^5^C regulators of DNA and RNA are upregulated, including NSUN3, TET2, DNMT2, ALYREF, DNMT3b, DNMT1, NOP2, and NSUN2. Furthermore, multiple m^5^C regulators were correlated with OS. NSUN4, NSUN7, DNMT1, DNMT3b, DNMT3a, NOP2, and NSUN5 were negatively correlated with OS, whereas NSUN6 was positively correlated with OS. Based on this, a prognostic model consisting of NSUN7, DNMT1, NSUN4, and NSUN6 was constructed [22].

In the human glioma cell line U87, NSUN2 mediates tumor cell migration by regulating the autotaxin (ATX)- lysophosphatidic acid (LPA) axis. NSUN2 methylates ATX mRNA 3’-UTR at cytosine 2756, thereby enhancing ATX mRNA translation. ATX-LPA pathway mediates the migration of cancer cells. Moreover, ALYREF interacts with methylated ATX mRNA to promote its export from the nucleus to cytoplasm. NSUN2-KO inhibits the migration of U87 cells, which can be recovered by the addition of LPA [72].

In the in vivo glioma models, NSUN5 showed hypermethylation of the CpG island promoter, leading to a reduction in transcripts and epigenetic silencing. NSUN5 silencing induced the deletion of 28S rRNA methylation at position C3782. The unmethylated state leads to the overall depletion of protein synthesis while activating the specific mRNA translation program under stress conditions, which results in the upregulation of NAD(P)H quinone dehydrogenase 1 (NQO1) protein. NQO1 overexpression confers sensitivity to drugs that target NQO1. Therefore, NSUN5 epigenetic silencing is a protective factor in gliomas and is correlated with a better prognosis [95].

### Breast cancer

In breast cancer cells and tissues, NSUN2 DNA hypomethylation leads to overexpression of NSUN2 mRNA and protein. Upregulation of NSUN2 promotes proliferation, migration, and invasion of breast cancer cells, whereas NSUN2-KO inhibits these processes [67]. In triple-negative breast cancer (TNBC), NSUN2 expression is upregulated thereby acting as a tumor-promoting factor, whereas NSUN6 is downregulated as a tumor suppressor. NSUN2 and NSUN6 affect tumorigenicity and the tumor immune microenvironment (TIM) of breast cancer [59]. Furthermore, the upregulation of NSUN2 and NOP2 mRNA was significantly associated with shorter disease-free survival in breast cancer patients [62].

Conversely, Li Chunlai et al. showed that NSUN6 promotes bone metastasis in breast cancer. HER3 is phosphorylated by tyrosine kinase (RTK)-like orphan receptor 1 (ROR1). NSUN6 is recruited by p-HER3 to methylate MST1, thus affecting the kinase activity of MST1 and activating YAP. The activation and accumulation of YAP in the nucleus stimulates the expression of target genes that correlate with tumor cell proliferation and bone metastasis [105].

### Urinary tumor

In urothelial carcinoma of the bladder (UCB), NSUN2 and m^5^C reader YBX1 are upregulated, which are positively correlated with T and N stages, the tumor grades of UCBs and poor disease-free survival of UCB patients. As described previously, NSUN2 introduces m^5^C into the 3'UTR of HDGF mRNA. YBX1 further recruits ELAV1 to stabilize m^5^C-modified mRNA to modulate the expression of HDGF. Invasion and metastatic abilities were significantly diminished in NSUN2- and YBX1-KO T24 cells [10]. As an oncogene in multiple cancers, HDGF has been shown to promote aggression and invasion [145–147].

In prostate cancer, the expression of NOP2 is elevated, which promotes metastasis and invasion through the EMT pathway [18]. NOP2 is the target gene of miR-PVT1 and miR-542-3p and is indirectly regulated by the lncRNA LINC00963 [18, 19]. Moreover, the level of DNMT2 is higher in tumor cells than in non-tumor epithelium, and in lymph node metastatic foci than in primary cancer. The expression of DNMT2 also increases in patients receiving androgen ablation therapy [148].

In clear cell renal cell carcinoma (ccRCC), the mRNA levels of NOP2 and NSUN4 are higher in tumor tissues than in normal tissues, whereas the mRNA levels of NSUN6 and m^5^C eraser TET2 are lower. The four m^5^C regulators constitute a risk signature for determining prognosis of patients [23]. High NOP2 expression in ccRCC was associated with poor OS [21]. Another study showed upregulation of NSUN5, ALYREF, DNMT3b, DNMT3A, NSUN2, NOP2, and DNMT1, and downregulation of NSUN3, NSUN4, NSUN7, and TET2 in ccRCC. The study proposed a risk signature consisting of seven m^5^C regulators: NOP2, NSUN2, NSUN3, NSUN4, NSUN5, TET2, and DNMT3b [20].

### Other cancers

In gallbladder carcinoma (GBC), the expression of NSUN2 is elevated in both cells and tissues. NSUN2 silencing inhibits the proliferation and tumorigenesis of GBC cells, whereas its overexpression promotes their growth. RPL6 modulates the translation of NSUN2 mRNA to exert carcinogenic effects. In RPL6 silenced cells, the level of NSUN2 protein was reduced, resulting in NSUN2 mRNA accumulation [149].

In lung squamous cell carcinoma (LUSC), NSUN3 and NSUN4 are upregulated and associated with poor prognosis. These are utilized to construct a prognostic risk signature. Furthermore, NSUN3 and NSUN4 are correlated with the infiltration of six major immune cells [79]. In lung adenocarcinoma, in vitro experiments indicated that cells with high expression of NOP2 or heterogeneous nuclear ribonucleoprotein (hnRNP) are more likely to be poorly differentiated [26]. Interestingly, loss of the region containing NSUN3 is common in non-smokers with lung adenocarcinoma at a frequency of 15% [150].

In cutaneous melanoma (CM), DNMT2, NSUN3, NSUN6, YBX1, and NOP2 are differentially expressed and used to calculate risk scores in patients. In particular, the upregulation of NOP2 and the downregulation of NSUN6 are closely associated with the progression of melanoma [24].

In esophageal squamous cell carcinoma (ESCC), NSUN2 is overexpressed and plays an oncogenic role [11, 50]. NSUN2 is known to be positively regulated by E2F transcription factor 1 (E2F1) and induces m^5^C modification in the 3'UTR of growth factor receptor-bound protein 2 (GRB2) mRNA. The Lin-28 homologous B (LIN28B) recognizes the modification to enhance GRB2 stability, through which elevated GRB2 activates PI3K/Akt and ERK/MAPK signaling [11]. Another study showed that NSUN2 methylated a novel lncRNA named NSUN2 methylated lncRNA (NMR). NMR promotes the metastasis and invasion of ESCC and enhances their resistance to cisplatin, possibly because m^5^C modified NMR inhibits the methylation of potential mRNAs [50].

In head and neck squamous cell carcinoma (HNSCC), the expression of NSUN2 is significantly upregulated, which correlates with shorter OS as well as the expression of cell cycle checkpoint-related genes [66]. NSUN2 may be regulated by Klotho (KL) where its low expression is positively correlated with the higher expression of KL and KL DNA hypomethylation [65]. Moreover, NSUN2 expression was negatively correlated with T-cell activation score. Higher mortality was observed in patients with low NSUN2 expression and high T cell activation scores [63].

In hypopharyngeal squamous cell carcinoma (HPSCC), mRNA and protein levels of NSUN2 are upregulated. NSUN2 modified 3'UTR of TEA domain transcription factor 1 (TEAD1) mRNA with m^5^C which promotes the expression of TEAD1, thereby enhancing the proliferation and invasion of tumor cells [71]. TEAD1 coordinates and integrates multiple signaling pathways. Its downregulation affects the expression of various oncogenes that modulate the progression, metastasis, and resistance of tumor cells to chemotherapy [151–153].

In pancreatic cancer (PC), the level of NSUN6 decreased significantly. Overexpression of NSUN6 inhibits the proliferation of PC cells and enhances CDK10 levels, suggesting that NSUN6 may regulate the growth of PC tumors by modulating CDK10. High expression of NSUN6 can predict lower risk and better prognosis in patients with PC [154].

### m^5^C RNA methyltransferases in cancer therapy

Although no specific inhibitor of m^5^C RNA methyltransferase has been developed thus far, several chemicals can interact with these methyltransferases to inhibit cancer progression. It has been reported that azacytidine can inhibit the methylation of C38 of tRNA^Asp^, catalyzed by DNMT2, to reduce the metabolic activity of cancer cells [155]. In breast cancer cells, the phytochemicals sulforaphane (SFN), ursolic acid (UA), and betulinic acid (BA) can reduce the expression of NOP2 and inhibit cell proliferation, possibly contributing to reduced translation efficiency caused by interference of ribosome formation [156].

m^5^C RNA methyltransferase also regulates drug resistance in cancer cells. In leukemia, RNA m^5^C enzymes regulate sensitivity and resistance to 5-Azacytidine (5-AZA). In 5-AZA-sensitive leukemia cells (ASLCs), NSUN3 and DNMT2 interact directly with hnRNP, which is involved in the formation of a 5-AZA-sensitive chromatin structure which forms a complex essential for the integrity of these proteins. In 5-AZA-resistant leukemia cells (ARLC), the interaction of NOP2, BRD4, and RNA pol-II is associated with the formation of an active chromatin structure with resistance to 5-AZA but is highly sensitive to the inhibition of BRD4 and NOP2 [81]. Moreover, NSUN2 and methyltransferase 1 (METTL1), another tRNA methyltransferase, enhance the cancer cell resistance to 5-fluorouracil (5-FU) by stabilizing tRNA and preventing RTD through methylation [157]. Notably, NSUN2 phosphorylation by Aurora-B led to its reduced enzymatic activity [158]. In glioblastoma, NSUN2 is a target gene of nuclear respiratory factor 1 (NRF1), and its high expression is associated with resistance to temozolomide (TMZ) therapy [64]. In melanoma, the increased expression of NSUN5 is used to predict the sensitivity of melanoma cells to the pyrazopyrimidine derivative c-Src inhibitor 10a [159].

DNMT2 also modulates the adverse effects on cancer cells associated with chemotherapy-induced senescence [160].

## Conclusion

In this review, we have summarized the molecular mechanisms and biological implications of m^5^C RNA methyltransferases and discussed their potential roles in cancer. m^5^C RNA methyltransferases are modifiers which introduce m^5^C into a variety of RNAs. In mRNAs, m^5^C modifications can modulate stability and mediate nuclear export and translation, while in ncRNAs, m^5^C modifications affects their stability, processing, cleavage, transcription, and translation. The downstream effects of these molecular functions/processes further mediate the regulation of various cellular functions, including cell proliferation, differentiation, migration, senescence, stress response, and inflammation. Interestingly, m^5^C RNA methyltransferase is also involved in the catalysis of m6A, which has a combinatorial effect with m^5^C. In conclusion, m5C methyltransferase is being recognized as a significant factor in post-transcriptional regulation because emergent studies on its regulatory mechanism, prognostic function, and target therapy are emphasizing its potential and feasibility for clinical application.

Although the functions of m^5^C RNA methyltransferase in cancer have become the focus of many studies in recent years, our knowledge is still far from complete. At present, no studies have discussed the interaction network between m^5^C methyltransferases, which may cause the regulatory mechanisms of key pathways in cancer to be neglected. Moreover, the specific function of some m^5^C sites, such as the methylation of tRNA by NSUN2 and NSUN6 and the methylation of 28S rRNA by NOP2, has not been determined. Notably, most current studies focus on mRNA, but the modification of rRNA by NSUN5 and the modification of lncRNA and miRNA by NSUN2 suggest the potential of m^5^C modification of non-coding RNA for cancer development. Furthermore, for RNA methyltransferases with multiple substrates, it is difficult to confirm which RNA modification causes phenotypic changes through single gene silencing experiments. More precise experimental designs are required to clarify their functions. In addition, the reader and eraser for m^5^C modifications should closely examined. Compared with the understanding of m^6^A modification, the current knowledge on m^5^C-related regulators is lacking, because it is hard to describe their biological processes and functions comprehensively. In mRNAs, m^5^C levels are lower (0.02–0.09%) [3] than m^6^A levels (0.4–0.7%) [161, 162], which entails the development of a more sensitive and reliable detection method for m^5^C. At present, none of the specific m^5^C RNA methyltransferase inhibitors have been developed as antitumor drugs.

Though studies of m^5^C RNA methyltransferases are helpful in revealing the mechanisms and roles of RNA methylation, a deep understanding of the pathogenesis and development of cancer becomes essential for efficient evaluation and treatment of patients. Based on the detailed review, we expect that upcoming studies on m^5^C RNA methyl transferases would address the following four aspects: (a) detecting the aberrant expression of m^5^C methyltransferases in cancers and constructing risk scores to assess patient survival; (b) exploring the targets of m^5^C RNA methyltransferases and constructing a regulatory crosslink model consisting of the associated molecular pathways; (c) developing targeted therapies related to m^5^C to provide new potential options for cancer treatment; and (d) developing high-precision and universal m^5^C detection sequencing techniques suitable for mRNAs.

## Data Availability

Not applicable.

## Abbreviations

m^5^C: 5-Methylcytosine
NSUN: NOL1/NOP2/sun
DNMT2: DNA methyltransferase 2
ALKBH: 1Alpha-ketoglutarate-dependent dioxygenase ABH1
TET: Ten-eleven translator family proteins
ALYREF: RNA and export factor-binding protein 2
f^5^C: 5-Formylcytosince
hm^5^C: 5-Hydroxymethylcytosine
YBX1: Y-box-binding protein 1
LIN28B: Lin-28 homologous B
SAM: S-adenosylmethionine
TERC: Telomerase RNA component
LTR: Long terminal repeat
NuSAP: Nucleolar and spindle-associated protein
IL-17A: Interleukin-17A
HHcy: Hyperhomocysteinemia
ICAM-1: Intercellular adhesion molecule-1
CDK1: Cyclin dependent kinase 1
UTR: Untranslated regions
5' tRFs: 5' TRNA-derived small RNA fragments
WBS: Williams-Beuren syndrome
CC: Corpus callosum
NMDAr: NMDA receptor
OPCs: Oligodendrocyte precursor cells
OFT: Outflow tract
TOF: Tetralogy of Fallot
CRC: Colorectal cancer
MST1: Macrophage stimulating 1
YAP: Yes-associated protein
PGC-1α: Peroxisome proliferator-activated receptor-gamma coactivator 1 alpha
HCC: Hepatocellular carcinoma
FZR1: Fizzy-related-1
GI: Gastrointestinal
OS: Overall survival
GC: Gastric cancer
SUMO: Small ubiquitin-like modifier
FOXC2: Forkhead box protein C2
EMT: Epithelial-mesenchymal transition
CDKN1C, p57^Kip2^: Cyclin-dependent kinase inhibitor 1C
PIK3R1: Phosphoinositide-3-kinase regulatory subunit 1
PCYT1A: Phosphate cytidylyltransferase 1A
GIST: Gastrointestinal stromal tumor
circRNAs: Circular RNAs
YTHDC1: YTH domain containing 1
HMGA2: High mobility group AT-hook 2
IGF2BP2: Insulin like growth factor 2 mRNA binding protein 2
LM: Liver metastasis
ROCK2: Rho associated coiled-coil containing protein kinase 2
ALO: Aloperine
PAR2: Protein activated receptor 2
Gab2: Grb associated-binding protein 2
ATX: Autotaxin
LPA: Lysophosphatidic acid
NQO1: NAD(P)H quinone dehydrogenase 1
TNBC: Triple-negative breast cancer
TIM: Tumor immune microenvironment
RTK: Receptor tyrosine kinase
ROR1: RTK-like orphan receptor1
UCB: Urothelial carcinoma of the bladder
HDGF: Heparin binding growth factor
ELAV1: ELAV like RNA binding protein 1
ccRCC: Clear cell renal cell carcinoma
GBC: Gallbladder carcinoma
LUSC: Lung squamous cell carcinoma
hnRNP: Heterogeneous nuclear ribonucleoprotein
CM: Cutaneous melanoma
ESCC: Esophageal squamous cell carcinoma
E2F1: E2F Transcription Factor 1
GRB2: Growth factor receptor-bound protein2
CSD: Cold shock domain
HNSCC: Head and neck squamous cell carcinoma
KL: Klotho
HPSCC: Hypopharyngeal squamous cell carcinoma
TEAD1: TEA Domain Transcription Factor 1
PC: Pancreatic cancer
SFN: Sulforaphane
UA: Ursolic acid
BA: Betulinic acid
5-AZA: 5-Azacitidine
ASLC: AZA-sensitive leukemia cells
ARLC: 5-AZA-resistant leukemia cells
METTL1: Methyltransferase 1
NRF1: Nuclear respiratory factor 1
